# Spatial and temporal analysis of tuberculosis incidence in Guinea-Bissau, 2018 to 2020

**DOI:** 10.1590/0034-7167-2022-0481

**Published:** 2023-10-09

**Authors:** Adelia Roberto Nanque, Antônio Carlos Vieira Ramos, Heriederson Sávio Dias Moura, Thaís Zamboni Berra, Reginaldo Bazon Vaz Tavares, Aline Aparecida Monroe, Ione Carvalho Pinto, Ricardo Alexandre Arcêncio

**Affiliations:** I Universidade de São Paulo. Ribeirão Preto, São Paulo, Brazil

**Keywords:** Tuberculosis, Spatial Analysis, Guinea-Bissau, Ecological Studies, Epidemiology, Tuberculosis, Análisis Espacial, Guinea-Bissau, Estudios Ecológicos, Epidemiología, Tuberculose, Análise Espacial, Guiné-Bissau, Estudos Ecológicos, Epidemiologia

## Abstract

**Objective:**

to analyze the epidemiological profile, spatial and temporal distribution of tuberculosis in Guinea-Bissau from 2018 to 2020.

**Methods:**

an ecological study, carried out in Guinea-Bissau, considering new cases of tuberculosis. Spatial analysis of areas was used to verify tuberculosis distribution in the country, and time series were used to identify incidence evolution over the years of study.

**Results:**

a total of 6,840 new cases of tuberculosis were reported. Tuberculosis incidence rate in the country ranged from 36.8 to 267.7 cases/100,000 inhabitants, with emphasis on the regions of Bissau and Biombo (over 90 cases/100,000). By using time series, it was possible to observe an increase in case incidence over the years of study.

**Conclusions:**

the study made it possible to identify the epidemiological profile of tuberculosis in Guinea-Bissau, spatial distribution heterogeneity, in addition to identifying the disease evolution over the years of investigation.

## RESUMO

**Objetivo:**

analisar o perfil epidemiológico, a distribuição espacial e temporal da tuberculose em Guiné-Bissau, no período de 2018 a 2020.

**Métodos:**

estudo ecológico, realizado em Guiné-Bissau, considerando os casos novos de tuberculose. Empregaram-se análise espacial de áreas, para verificar a distribuição da tuberculose no país, e séries temporais, para identificar a evolução da incidência ao longo dos anos de estudo.

**Resultados:**

foram notificados 6.840 casos novos de tuberculose. A taxa de incidência da tuberculose no país variou de 36,8 a 267,7 casos/100.000 habitantes, com destaque para as regiões de Bissau e Biombo (superior a 90 casos/100.000). Com o uso de séries temporais, foi possível observar um aumento na incidência dos casos ao longo dos anos do estudo.

**Conclusões:**

o estudo permitiu identificar o perfil epidemiológico da tuberculose em Guiné-Bissau, a heterogeneidade da distribuição espacial, além de identificar a evolução da doença ao logo dos anos de investigação.

## RESUMEN

**Objetivo:**

analizar el perfil epidemiológico, distribución espacial y temporal de la tuberculosis en Guinea-Bissau, de 2018 a 2020.

**Métodos:**

estudio ecológico, realizado en Guinea-Bissau, considerando nuevos casos de tuberculosis. Se utilizó análisis espacial de áreas para verificar la distribución de la tuberculosis en el país y series de tiempo para identificar la evolución de la incidencia a lo largo de los años de estudio.

**Resultados:**

se notificaron 6.840 nuevos casos de tuberculosis. La tasa de incidencia de tuberculosis en el país varió de 36,8 a 267,7 casos/100.000 habitantes, con énfasis en las regiones de Bissau y Biombo (más de 90 casos/100.000). Con el uso de series de tiempo, fue posible observar un aumento en la incidencia de casos a lo largo de los años del estudio.

**Conclusiones:**

el estudio permitió identificar el perfil epidemiológico de la tuberculosis en Guinea-Bissau, la heterogeneidad de la distribución espacial, además de identificar la evolución de la enfermedad a lo largo de los años de investigación.

## INTRODUCTION

Tuberculosis is an infectious disease associated with living conditions, with poverty being one of the main determining factors for the illness or worsening of its symptoms^(1)^. Housing conditions, food, per capita income, education, age, unemployment, overcrowding, difficulties in accessing health services, comorbidities and precarious sanitary conditions are the main socioeconomic indicators related to disease occurrence^(2-3)^.

Until 2019, tuberculosis was the main cause of death from a single infectious agent, a scenario that changed from 2020 onwards, with the advent of the COVID-19 pandemic. The COVID-19 pandemic brought negative impacts on global and national health systems, impairing actions for tuberculosis prevention, diagnosis and treatment, especially in countries with a high burden of the disease. According to the World Health Organization (WHO), between 2019 and 2020, there was an 18% reduction in tuberculosis diagnosis in the world, from 7.1 million (2019) to 5.8 million (2020), with a small recovery in 2021 (6.4 million)^(4-5)^.

Despite the pandemic context still present, it should be noted that tuberculosis remains a serious public health problem worldwide, especially in developing countries, such as Guinea-Bissau, who experience extreme socioeconomic inequality, due to constant political and institutional instability, in addition to having a fragile health system in terms of infrastructure, equipment, inputs and quality of care^(6)^.

According to the WHO report produced in 2022, Guinea-Bissau is on the list of nations with a high tuberculosis incidence, with a rate greater than 50 cases per 100,000 inhabitants in 2021^(7)^. According to the country’s Ministry of Public Health (MPH), the incidence (new cases and relapses) of tuberculosis increased from 305 to 361 cases per 100,000 inhabitants between 2000 and 2005, remaining stable until 2018. Regarding the tuberculosis mortality rate, in 2018, there were 72 deaths per 100,000 inhabitants, and in people living with HIV, the rate was 73 deaths per 100,000 inhabitants^(8)^.

To face the problem of tuberculosis in the country, the National Strategic Plan 2020-2024 was prepared, built according to the End TB Strategy indicators, principles and pillars^(9-10)^. Considering the plan’s guidelines and objectives, MPH states that co-infection with HIV is one of the greatest challenges in the fight against tuberculosis in the country, the number of cases of tuberculosis associated with HIV has increased dramatically in recent years, as tuberculosis is the most common opportunistic infection in people living with HIV/AIDS^(8)^.

Moreover, the low availability of diagnostic resources (inputs), underreporting of cases, low accessibility to health services, social stigmatization, poverty, difficulties in following up people undergoing treatment, lack of availability of data on the disease, lack of access to technologies (internet, computers, etc.), difficulties in systematizing care and difficulties in managing patients and data records are the main challenges related to combating tuberculosis in Guinea-Bissau^(11-12)^.

It also highlights the fragility in the management of pharmacies and the availability of medications in the country, verifying serious deficiencies and shortages resulting from a low level of forecasting and mastery of the entire supply chain of medications, shortages in the stock of important drugs and supplies for the diagnosis and treatment of various diseases, with an emphasis on tuberculosis and HIV/AIDS^(13-14)^.

One of the main situational and epidemiological diagnostic strategies applied in the context of tuberculosis refers to spatial and temporal analysis tool use, such as geoprocessing and time series, which contribute to the identification and understanding of disease transmission dynamics, providing evidence of priority areas or zones for control and intervention activities^(14-15)^. In a literature review, used for this research, no studies were found analyzing the situation of tuberculosis in Guinea-Bissau considering spatial and temporal analysis technique use so that the present research will be the first to study the disease in the country using these tools.

Given the magnitude of the problem, it is necessary to know the epidemiological situation of tuberculosis in Guinea-Bissau, which will contribute to the construction of specific strategies, in addition to the creation and implementation of public policies aimed at confronting this disease.

## OBJECTIVE

To analyze the epidemiological profile, spatial and temporal distribution of tuberculosis in Guinea-Bissau from 2018 to 2020.

## METHODS

### Ethical aspects

The study was conducted in accordance with national and international ethical guidelines. Ethical review and approval were waived because it was research that used secondary data and international public domain. Data were collected from the national information system District Health Information Software (DHIS2), and in Guinea-Bissau there is no specific norm or law regarding secondary data use.

### Study design, period and place

**Figure 1:**
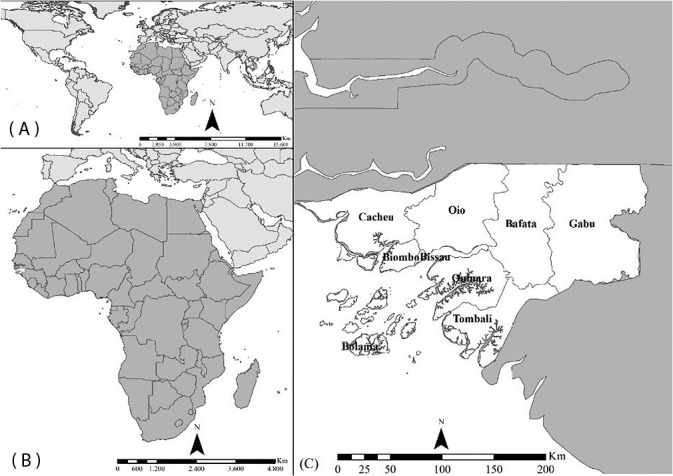
Guinea-Bissau location map

This is an ecological study^(16)^ carried out in Guinea-Bissau from 2018 to 2020. Guinea-Bissau is a Portuguese-speaking African Country (PSAC) located on the west coast of Africa. Guinea-Bissau has a territorial extension of 36,125 km^2^, consisting of a continental part that covers 78% of its territory, and an insular part, called the Bijagós Archipelago, composed of 88 islands and an estimated population, in 2020, of 1,959,139 inhabitantss^(17)^. The country is divided into nine administrative regions, namely: Bafatá, Biombo, Bolama, Cacheu, Gabú, Oio, Quinara, Tombali, and the capital Bissau (Figure 1).

*Caption: (A) World map; (B) Africa; (C) Administrative districts of Guinea-Bissau.*

*Source: District Health Information Software, Guinea-Bissau, 2021.*

Guinea-Bissau’s health system brings together the public health service, the private sector and traditional medication. The public health system has a pyramid shape and comprises three levels: peripheral (local), regional and central. The peripheral level includes health centers and health centers of type A (hospitals, rural), B (health centers, rural) and C (basic health centers, urban). The regional level is composed of regional hospitals and the central level has two national hospitals that are reference for tuberculosis treatment, *The Raoul Follereau Hospital*, located in the Autonomous Sector of Bissau, and *The Cumura Hospital*, located in the Biombo Region^(18)^.

Tuberculosis case diagnosis in Guinea-Bissau is carried out in accordance with the guidelines of the country’s National Program to Fight Tuberculosis. Diagnosis and follow-up activities for people with tuberculosis at the peripheral level are carried out by diagnosis and treatment centers, treatment centers and community health workers (CHW)^(17-18)^. It is noteworthy that until 2020, active search actions for tuberculosis cases were not carried out in the community, and from 2021, community health workers in the country became responsible for these actions.

Tuberculosis treatment in the country follows the scheme recommended by the WHO (rifampicin, isoniazid, pyrazinamide and ethambutol (two-month intensive phase), rifampicin and isoniazid (four-month maintenance phase)), carried out on an outpatient basis and in Basic Health Units.

In cases of complications and drug resistance, the reference units for disease treatment in the country (*Hospital Raoul Follereau* and *Hospital de Cumura*) receive patients referred by other health structures and are only submitted to treatment during the intensive phase (first two months) so that the remaining four months are carried out on an outpatient basis, and these people are counter-referred to their area of residence, where they must continue the treatment in the maintenance phase^(18)^.

### Population, inclusion and exclusion criteria

The study population consisted of new cases of tuberculosis reported in the DHIS2 system between 2018 and 2020. It should be noted that DHIS2 is an information system that has limitations in its structure and organization, and in the process of reporting the case of tuberculosis only information relating to the fields “new cases”, “relapses”, “clinical form”, “type of diagnosis”, “sex” and “age group” is fed. It should also be noted the unavailability of data prior to 2018, a factor that determined the choice of period for this research.

Data were collected in October 2021 through a request letter sent via email to the MPH. The variables related to health regions, sex, age group (only 2019 and 2020 considered), type of diagnosis, clinical form (pulmonary tuberculosis and extrapulmonary tuberculosis) and co-infection (HIV) were collected.

For the study construction, the EQUATOR network guidelines were followed, through the instrument STrengthening the Reporting of OBservational studies in Epidemiology (STROBE).

### Data analysis

After analyzing database record consistency, exploratory analyzes were carried out to characterize the profile of the cases. This step was performed using descriptive statistics of the quantitative parameters, the absolute and relative frequencies of variables health regions, sex, age group (2019 and 2020), type of diagnosis, clinical form and co-infection were calculated, using Microsoft Excel.

For the spatial analysis stage, the administrative regions of Guinea-Bissau were considered (analysis by area), with the annual incidence rate (2018, 2019 and 2020) calculation for each unit of analysis. The absolute number of new cases of tuberculosis (pulmonary and extrapulmonary) was considered in the numerator and the population of each administrative region in the denominator, with a multiplication factor per 100,000 inhabitants. GeoDa software version 1.20 was used to calculate the rates and ArcGIS 10.8 software to prepare choropleth maps.

After spatial analysis, time series^(19)^ of tuberculosis incidence rates in the country and for the nine administrative regions were constructed, with the intention of verifying incidence evolution over the years of study. Time series and their respective graphs were built in RStudio software, version 4.2.0.

## RESULTS

From 2018 to 2020, 6934 cases of tuberculosis were reported in Guinea-Bissau, of which 6840 were new cases of the disease. The highest percentage occurred in the capital Bissau (53%), corresponding to more than half of the cases. Males accounted for 63% of reported cases and the age group from 25 to 34 years old corresponded to 29% of the total.

Regarding the type of diagnosis, 83% (5654 cases) were diagnosed bacteriologically. Regarding the clinical profile, 97% of cases were of pulmonary tuberculosis, with coinfection cases representing 31% of the total (Table 1).

**Table 1:** Distribution of tuberculosis cases according to administrative regions and sociodemographic and clinical variables. Guinea-Bissau, 2018-2020

Variables	Cases	%
Regions		
Bafafá	624	9
Biombo	435	6
Bissau	3631	53
Bolama	70	1
Cacheu	508	7
Gabú	374	5
Oio	754	11
Quinara	176	3
Tombali	268	5
Sex		
Male	4308	63
Female	2532	37
Age group^*^		
0 to 4 years	88	2
5 to 14 years	163	3
15 to 24 years	895	18
25 to 34 years	1449	29
35 to 44 years	1230	25
45 to 54 years	700	14
55 to 64 years	391	8
≥65 years	85	1
Type of diagnosis		
Bacteriological	5654	83
Clinical	992	15
Others	194	2
Clinical form		
Pulmonary	6646	97
Extrapulmonary	194	3
Co-infection/HIV	1365	31

Fonte: District Health Information Software, Guiné-Bissau, 2021.

* Para variável faixa etária, foram considerados os anos de 2019 e 2020.

During the study period, the incidence rate of tuberculosis in Guinea-Bissau ranged from 36.8 to 267.7 cases per 100,000 inhabitants among the administrative regions, evidencing a pattern of heterogeneous disease spatial distribution in the country. The regions with the highest incidence can be considered priority locations for health actions and interventions.

**Figure 2:**
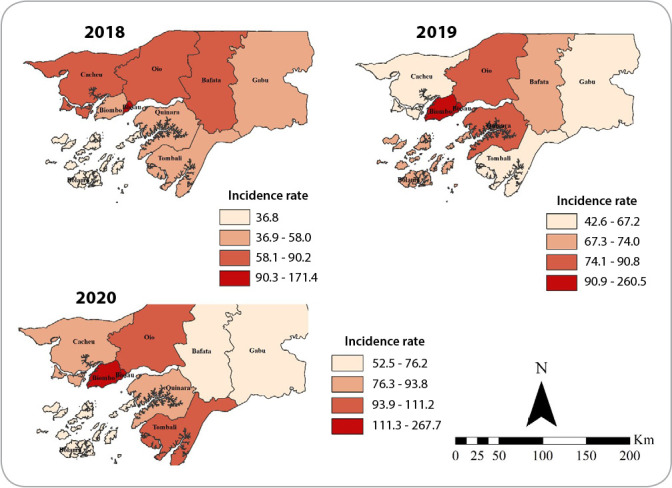
Spatial distribution of tuberculosis incidence according to administrative regions. Guinea-Bissau, 2018-2020

*Source: District Health Information Software, Guinea-Bissau, 2021.*

A distinct profile can be seen between years, in addition to an evolution of tuberculosis for all administrative regions. It can be seen that in 2018 the capital Bissau was the only one with an incidence greater than 90 cases per 100,000 inhabitants, but between 2019 and 2020, the regions of Biombo, Oio and Tombali showed an increase in disease incidence (Figure 2).

**Figure 3:**
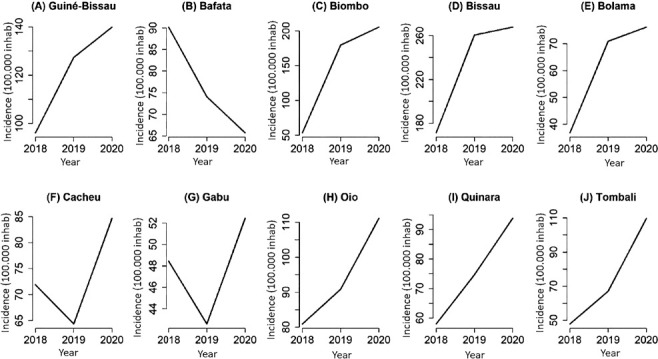
Time series of tuberculosis incidence rates according to administrative regions. Guinea-Bissau, 2018 to 2020

*Source: District Health Information Software, Guinea-Bissau, 2021.*

In Figure 3, it is possible to verify the time series of tuberculosis incidence rates per 100,000 inhabitants according to the administrative regions of Guinea-Bissau, over the years 2018 to 2020. An increase in the incidence over the years can be seen in almost all regions, including the country as a whole, with the exception of the Bafata region, which in the last two years analyzed (2019 and 2020) showed a drop in incidence rate. It should be noted that the regions of Cacheu and Gabú also showed a drop in incidence in 2019, with a subsequent increase in 2020. It is worth noting that the capital Bissau had an incidence rate of 180 cases per 100,000 inhabitants in 2018, rising to 260 cases per 100,000 inhabitants in 2020.

## DISCUSSION

This study aimed to analyze the epidemiological profile, the spatial and temporal distribution of tuberculosis in Guinea-Bissau from 2018 to 2020. With descriptive analysis referring to reporting by administrative region, it was identified that the highest percentage of tuberculosis cases was observed in males, aged between 25 and 34 years, including the economically active population, corroborating other investigations^(20-21)^. The reasons for the predominance of tuberculosis in male and young people may be associated with biological factors, lifestyles and lack of self-care, in addition to the fact that men, especially young men, seek less and/or belatedly for health services in comparison to women^(22-23)^.

Furthermore, in the present study, 15% of cases were clinically diagnosed, probably due to the lack of inputs for tuberculosis laboratory diagnosis in Guinea-Bissau, which often ends up being done clinically. It is known that the diagnosis using clinical score is an important tool for early identification of pulmonary tuberculosis in primary care, especially in places with low prevalence of HIV infection^(24)^. However, in the present study, co-infection with HIV represented 31% of cases, with a high incidence between the years analyzed, which can become an obstacle to the clinical diagnosis, using only individuals’ clinical forms.

Previously, in Guinea-Bissau, only the bacteriological method (direct sputum examination and culture) was used to diagnose tuberculosis. However, as of 2015, the molecular test began and, currently, the country has only nine GeneXpert devices for its performance, two of which are located at the National Public Health Laboratory (in the capital Bissau), two in the Main Military Hospital (in Bissau) and the others are uniformly distributed among several hospitals and reference centers throughout the country. In general, these devices are not enough and cannot meet the health system’s demand, causing a large number of people to be diagnosed by clinical assessment criteria, without bacteriological confirmation^(17-18)^.

The pulmonary form was predominant in this study, the percentage observed being higher than that found in other African countries, such as Morocco^(25)^, with pulmonary tuberculosis being present in 63.50% of cases, while 36, 50% of people diagnosed had extrapulmonary tuberculosis.

Pulmonary tuberculosis, in addition to being the most frequent form, is also the most relevant for public health services, as this form is responsible for maintaining the disease transmission chain. Thus, the active search for individuals with respiratory symptoms is the main strategy for controlling tuberculosis, as it allows for early detection and rapid initiation of treatment for pulmonary forms^(26)^.

A total of 31% of cases with HIV co-infection were observed, a percentage higher than that found in a study carried out in Ethiopia (7.34%)^(15)^, and lower than that found in Zimbabwe (72%)^(27)^ and Uganda (47%)^(28)^.

Co-infection with HIV represents a major challenge in the fight against tuberculosis globally, especially in African countries, as it represents the main burden of infectious diseases in countries with limited resources, with HIV co-infection being the best known and most powerful risk factor for progression of *Mycobacterium tuberculosis* infection to the active form of the disease, in addition to increasing the risk of reactivation of latent tuberculosis by up to 20 times^(29)^.

The HIV epidemic in Guinea-Bissau has a national prevalence of 3.3%, and data resulting from epidemiological projections indicate that more than 30,000 people are living with HIV in the country, most of them adults. In 2014, the total number of people living with HIV in Guinea-Bissau was 35,997, with the overall prevalence being slightly higher in urban areas (3%) than in rural areas (2.3%), and higher in women (5%) than in men (1.5%)^(11)^.

The poor communication between tuberculosis and HIV programs in the country directly affects quality of care and patient control, needing to be improved to achieve a better quality of care, in the provision of services to users and, especially, in the production of reliable data on the epidemiological situation of tuberculosis and its co-infection with HIV in the country. It should be noted that the active search and HIV diagnosis are also negatively influenced by irregular input supply for carrying out diagnostic tests, which can influence disease underreporting, in addition to the fact that the country also has insufficient access to antiretrovirals, further aggravating the health situation in Guinea-Bissau^(11)^.

The overall incidence rate during the investigation period was 140 cases per 100,000 inhabitants, considered “high” according to WHO parameters. According to the Global Tuberculosis Report 2022, the African continent is the region with the highest incidence rates of tuberculosis in the world, contemplating 16 countries from the list of the 30 priority nations for disease control. Although Guinea-Bissau is not on this list, it should be noted that it is a nation with one of the highest numbers of cases of tuberculosis and co-infection with HIV on the continent^(7)^.

Both spatial analysis and time series analysis showed that the capital Bissau is the region most affected by tuberculosis in the country, with an incidence rate greater than 250 cases per 100,000 inhabitants, followed by the region of Biombo, with an incidence rate of 200 cases per 100,000 inhabitants. This similarity in the epidemiological profile between the two regions is probably due to their geographic proximity and internal migration, which allows for greater contact between people and greater circulation of the bacillus between the regions^(30)^.

It was also possible to show that Bissau, the region with the highest population density and the highest urbanization rate in the country, offered a higher risk for the transmission of tuberculosis, a result similar to that found in other studies^(2,12,15)^. Highly populated urban areas are vulnerable places that offer greater conditions for transmission of tuberculosis, due to the influence of social factors such as poor housing conditions, high number of residents per household, high population density, lack of basic sanitation, insufficient means of transport that generate crowds, lack of urban infrastructure and difficulties in accessing quality education and health services^(2,17,31)^.

According to a survey carried out by the National Institute of Statistics of Guinea-Bissau, carried out in 2018, regarding employment issues, similar to all African capitals, Bissau was the main region of installation of internal migrants (23.2%) as well as such as the Biombo region (15.5%)^(32)^. Since they are cities that concentrate most of the country’s infrastructure (health, education, employment, justice, transport, among other services), they attract people who seek better access to reference urban facilities and the job market. These characteristics influence the uncontrolled growth and rapid urbanization of these regions, which may explain the increased circulation and spread of *Mycobacterium tuberculosis* in these places.

During the period studied, the temporal trend of tuberculosis incidence in Guinea-Bissau and in most administrative regions was increasing, probably influenced by the increase in HIV cases^(33)^, in addition to the characteristics of extreme poverty found in the country. According to the National Institute of Statistics of Guinea-Bissau, almost 765 thousand people live in poor households, representing 64.7% of the total population, which means that almost two thirds of Guineans are affected by poverty^(32)^.

In addition to these alarming data, people affected by extreme poverty number more than 245,000, corresponding to an incidence of 20.8% of the population. The classification of administrative regions, according to poverty incidence and the contribution of each one to poverty at national level, follows the following order: Oio (79.6% and 18.0%), Bafatá (72.4% and 13.6%), Quinara/Tombali (69.1% and 12.2%), Gabú (65.8% and 12. 3%), Cacheu (63.8% and 14.2%), Biombo/Bolama (62.6% and 9.1%) and Bissau (51.6% and 20.6%)^(34)^.

In this way, it is emphasized that tuberculosis and poverty have a bidirectional relationship, tracing a profile that makes people live in a situation of social vulnerability, because both poverty can be related to precarious health conditions and these can also produce poverty, limiting opportunities for work and subsistence^(35)^.

One aspect worth mentioning refers to the possible effect of the COVID-19 pandemic on tuberculosis incidence rates in Guinea-Bissau. According to WHO data^(36)^, comparing the years 2019 and 2020, more than 200 countries showed significant reductions in tuberculosis reporting, with drops of 25 to 30%. This reduction in reporting, among other factors, was due to the interruption/reorganization of national health services to face COVID-19, with medium and long-term repercussions, with estimates of an increase in the number of cases and deaths from tuberculosis in the coming years.

In the present research, the investigation period included the first year of the pandemic (2020) so that, despite the high rates identified (nationally and by administrative region), the hypothesis of possible underreporting of tuberculosis cases that year in Guinea-Bissau is raised so that the situation of the disease in the country may be even more serious than that found in this study.

The study advances knowledge by generating information that can support the formulation and implementation of strategies to combat tuberculosis in Guinea-Bissau, generating information that contributes to the National Tuberculosis Fight Program, in addition to identifying that social inequality is one of the main obstacles to fighting the disease in the country.

### Study limitations

The study has limitations, with emphasis on the difficulty in accessing data, the lack of cartographic bases of the health regions of Guinea-Bissau, in addition to the low quality of the data collected, with failures in filling, for instance, no record of closure and socioeconomic situations of people diagnosed. Moreover, it is worth highlighting the fact that this is an ecological study, and therefore, its results cannot be generalized to the individual level.

Despite the limitations regarding the data used, the findings showed the magnitude of the problem and allowed assessing the epidemiological situation of tuberculosis in Guinea-Bissau, enabling the identification of areas with the highest risk for tuberculosis transmission occurrence, in addition to identifying the evolution of the disease over time.

### Contributions to nursing, health, and public policies

The results allowed knowing the difficulties faced by Guinea-Bissau in coping with tuberculosis from 2018 to 2020. As it is a disease of a social nature, it is of fundamental importance to identify the regions with the highest incidence, for the planning of actions with a view to combating and elimination of the disease, in line with the WHO End TB strategy assumptions.

Knowledge of the clinical and epidemiological profile of tuberculosis and its spatial and temporal distribution will allow better planning of public policies, execution, monitoring and assessment of health services’ actions in the control of this disease, with the consequent reduction of the population’s risk and suffering, visualizing the reduction and elimination of the disease in the country.

As contributions to public health, the present study will raise the awareness of government officials about the problem of tuberculosis in Guinea-Bissau, which may imply public policies for managing tuberculosis and improving the population’s living conditions. As contributions to nursing, the study will help nurses to have a broad view of tuberculosis in the country, especially in management activities for tuberculosis surveillance and control, in the planning of health intervention strategies, serving as a subsidy for discussion with professionals health, community, students and government officials on the subject.

## CONCLUSIONS

The study made it possible to highlight the epidemiological profile of tuberculosis, the magnitude of the problem and spatial distribution heterogeneity, identifying areas with greater risk for disease occurrence, in addition to elucidating its evolution over time. The present investigation allowed the raising of hypotheses about fighting tuberculosis in the country, highlighting the need for investments in public policies in research, in health professional training, in health education actions, in addition to reinforcing surveillance actions (active search for respiratory symptoms in the community). Finally, it is important to point out that through strategies related to early diagnosis, monitoring, assessment and follow-up of patients with tuberculosis, it is possible to think about reducing the high tuberculosis incidence rate in Guinea-Bissau.
